# Polyurethane Culture Substrates Enable Long-Term Neuron Monoculture in a Human *in vitro* Model of Neurotrauma

**DOI:** 10.1089/neur.2023.0060

**Published:** 2023-10-16

**Authors:** Angela Mitevska, Citlally Santacruz, Eric J. Martin, Ian E. Jones, Arian Ghiacy, Simon Dixon, Nima Mostafazadeh, Zhangli Peng, Evangelos Kiskinis, John D. Finan

**Affiliations:** ^1^ Department of Biomedical Engineering, University of Illinois at Chicago, Chicago, Illinois, USA.; ^2^ The Ken & Ruth Davee Department of Neurology, Feinberg School of Medicine, Northwestern University, Chicago, Illinois, USA.; ^3^ Department of Mechanical and Industrial Engineering, University of Illinois at Chicago, Chicago, Illinois, USA.; ^4^ Biomer Technology Ltd., Warrington, United Kingdom.; ^5^ Department of Neuroscience, Feinberg School of Medicine, Northwestern University, Chicago, Illinois, USA.

**Keywords:** finite element models, *in vitro* studies, models of injury, stem cells

## Abstract

Human induced pluripotent stem cell (hiPSC)-derived cells can reproduce human-specific pathophysiology, patient-specific vulnerability, and gene-environment interactions in neurological disease. Human *in vitro* models of neurotrauma therefore have great potential to advance the field. However, this potential cannot be realized until important biomaterials challenges are addressed. *Status quo* stretch injury models of neurotrauma culture cells on sheets of polydimethylsiloxane (PDMS) that are incompatible with long-term monoculture of hiPSC-derived neurons. Here, we overcame this challenge in an established human *in vitro* neurotrauma model by replacing PDMS with a highly biocompatible form of polyurethane (PU). This substitution allowed long-term monoculture of hiPSC-derived neurons. It also changed the biomechanics of stretch injury. We quantified these changes experimentally using high-speed videography and digital image correlation. We used finite element modeling to quantify the influence of the culture substrate's thickness, stiffness, and coefficient of friction on membrane stretch and concluded that the coefficient of friction explained most of the observed biomechanical changes. Despite these changes, we demonstrated that the modified model produced a robust, dose-dependent trauma phenotype in hiPSC-derived neuron monocultures. In summary, the introduction of this PU film makes it possible to maintain hiPSC-derived neurons in monoculture for long periods in a human *in vitro* neurotrauma model. In doing so, it opens new horizons in the field of neurotrauma by enabling the unique experimental paradigms (e.g., isogenic models) associated with hiPSC-derived neurons.

## Introduction

Neurotrauma remains a major cause of mortality and morbidity with poorly understood mechanisms and few treatment options. In 2014, the Centers for Disease Control and Prevention documented 2.53 million emergency unit visits, approximately 288,000 hospitalizations, and 56,800 deaths related to traumatic brain injury (TBI).^1^ Spinal cord injury (SCI) affects 1.3 million North Americans, with more than half of the cases being post-traumatic.^2^ TBI increases the long-term risk of Alzheimer's disease,^3^ Parkinson's disease,^4^ amyotrophic lateral sclerosis (ALS),^5^ and all causes of dementia.^6^ Patient genotype also influences outcomes,^7^ and genetic risk factors may synergize with the risks posed by TBI.^8,9^ Environmental and genetic risk factors pose clinical challenges, but they also present scientific opportunities. Studies of these factors may ultimately reveal molecular mechanisms of pathology and lead to novel therapeutic targets. However, exploring genetic and environmental risk factors and the interactions between them is difficult in clinical studies and pre-clinical animal models. Clinical subjects vary widely in terms of their demographics, their genetics, and the biomechanics of the injury event. Pre-clinical animal models cannot precisely reproduce patient genotype. Human *in vitro* models can address some of these challenges.

Human induced pluripotent stem cells (hiPSCs) can be generated from patients of any genetic background and differentiated into many types of terminal cells.^10^ They retain the genome of the donor patient and allow it to be edited precisely to test hypotheses about individual genetic variants.^11^ Human *in vitro* models reproduce important pathological mechanisms in neurological diseases such as bipolar, ALS and Parkinson's disease.^12–15^ Human *in vitro* models have also been applied to neurotrauma using stretchable cell-culture substrates.^16^ These models reproduce many important acute injury phenotypes, including cell death, mitochondrial dysfunction, and neurite degeneration. However, they are limited to acute phenotypes because hiPSC-derived neurons cannot survive for long periods on *status quo* stretchable cell-culture substrates.

Stretch injury models are the most popular type of *in vitro* neurotrauma model.^17^ In these models, neural cells are grown on stretchable substrates and the substrate is stretched using a pulse of air pressure or a rigid indenter.^18^ In *status quo* stretch injury models, the stretchable cell-culture substrate is composed of polydimethylsiloxane (PDMS).^19,20^ PDMS has a long history of successful application in cell biomechanics; its elasticity, optical transparency, and ease of manufacture make it a popular material in the field.^21^ However, PDMS is also mildly neurotoxic, so it is difficult to sustain long-term monocultures of neurons on this substrate.^22,23^ PDMS also disrupts endocrine function and it absorbs drugs, thereby altering the drug concentration in the cell-culture media.^21,24,25^ These properties of PDMS frustrate investigations of trauma pathology in human *in vitro* models. The goal of this study is to identify an alternative cell-culture substrate that enables long-term culture of induced pluripotent stem cell (iPSC)-derived neurons in an *in vitro* neurotrauma model. We test the hypothesis that appropriately prepared polyurethane (PU) elastomer improves biocompatibility while retaining the mechanical properties necessary for this demanding application.

## Methods

### Polyurethane and polydimethylsiloxane plate fabrication

We fabricated the PU plates as follows: We cut out a piece of double-sided microfluidic diagnostic tape (3M; catalog number: 7100067080) with a hole pattern matching the bottom of a 96-well plate using a Silhouette Portrait 2 Cutting Machine (Silhouette America Inc.). We attached a PU (Biomer Technology Ltd.) sheet to the underside of a bottomless plate (Nunc) using this tape. We fabricated PDMS plates as previously described.^16^ Briefly, we plasma-treated the plate bodies, immersed them in (3-aminopropyl)triethosiloxane, rinsed them in water, and then dried them with compressed air. We then stretched PDMS membranes (Specialty Manufacturing Inc.) with a custom-built frame, plasma-treated them in a plasma cleaner, and clamped them to the bottomless plates, causing a covalent bond to form between the plate body and the PDMS.

### Plate indentation

**FIG. 1.**
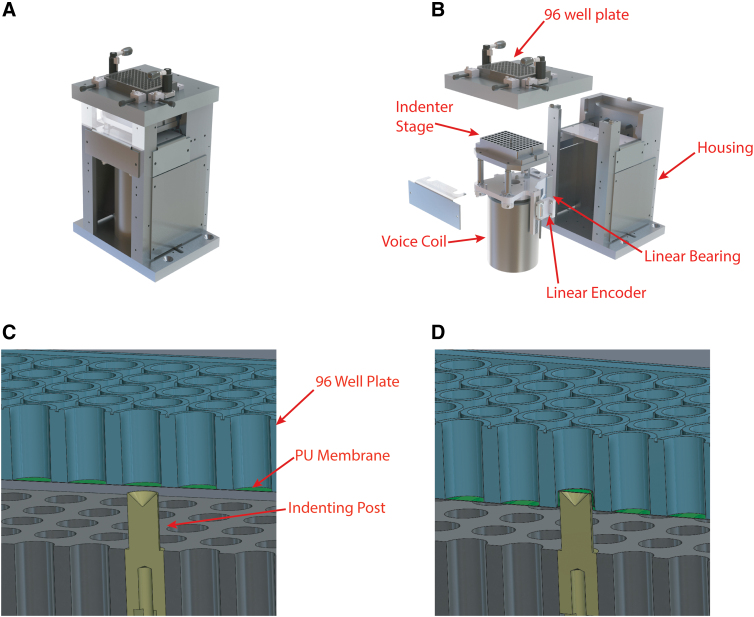
Injury device. (**A**) Complete apparatus. (**B**) Exploded view showing key components. (**C**) Cross-section view of a single post at the initial position for the injury procedure. (**D**) Cross-section view of a single post at peak indentation depth (panels A and B are reproduced with permission^16^).

We used a plate indentation device designed and built in-house to apply repeatable, biofidelic strains and strain rates to the well bottoms (Fig. 1A). This device is an updated version of a similar device that was described in detail in previous publications.^16,23,26^ Briefly, it consists of a stationary platform that supports the 96-well plate under which sits a LA43-67-000A voice coil (Sensata Technologies) that drives a stage up and down on a vertical linear bearing. The stage supports an aluminum block with an array of holes on the top surface that matches the positions of the wells in the 96-well plate. Teflon-coated vertical cylindrical posts can be mounted individually in these holes in any configuration (Fig. 1B). A T1031-30A linear quadrature encoder (Renishaw PLC) monitors the position of the stage and feeds it back to a Xenus XPL-230-40 servo drive (Copley Controls) controlled by a combination of the manufacturer's software (CME2) and custom-written python scripts.

To create injury, we lubricated the post array with corn oil, brought it to a position 1 mm below the bottom of the 96-well plate, and then displaced it up and down in a motion pulse with a period of 30 ms and an amplitude of 2–4 mm, with larger amplitudes creating more severe injuries (Fig. 1C,D). We chose these distance and duration values based on cadaveric studies of human TBI that tracked the extent and duration of brain strains with high-speed x-ray videography.^27^ The primary difference between this device and previously published versions is that this device holds the plate stationary and moves the post array up into it whereas previous versions held the post array stationary and moved the plate down on to it.^16^

### Membrane strain quantification

We used high-speed imaging and digital image correlation to quantify the amount of strain in the well bottom for a particular depth of indentation. Before indentation, we airbrushed the plates with black ink to create a speckled pattern on the top side of the membranes. We indented them as described above and imaged the well bottom at 1500 frames per second and 1024 × 1024 resolution using a FastCam High Speed Digital Camera (Photron) and a diffuse axial light. We quantified membrane strain in these images using GOM Correlate (Carl Zeiss GOM Metrology), a digital image correlation program. We exported the vertical and horizontal Green strain values as .csv files. The strain field was approximately equibiaxial with negligible shear, so we computed the maximum principal Green strain as the average of the X and Y Green strain.

### Polyurethane film production

We prepared PU films 250 mm long and 325 mm wide by film casting, utilizing a K Control Coater with a wire-bound bar applicator (RK PrintCoat Instruments Ltd., Royston, UK) onto a single-side, silicone-coated polyester (75 μm) release liner (HiFi Polyester Film, Stevenage, UK). We prepared an 8% (w/w) solution of biocompatible-grade PU pellets (ZYTAR™ Z1A1; Biomer Technology Ltd, Runcorn, UK) in n,n-dimethylacetamide (Sigma-Aldrich Co. Ltd, Gillingham, UK). We dispensed the polymer solution onto the release liner behind the bar applicator and drew the bar at a rate of 2 m/min to create a uniform coating. We placed the coated liner in a fan-controlled oven (Townsen Mercer Ltd., Altrincham, UK) equipped with a local exhaust venting unit (BOFA International Ltd., Poole, UK) at 89℃ for 60 min to remove the solvent. We allowed the dry film to cool for 30 min and then sealed it within the inner and outer PU package.

### Membrane thickness quantification

We placed samples of PDMS and PU film on glass slides and measured their thickness using a Tencor P-7 Stylus Contact Profilometer. This instrument pre-loaded a titanium stylus tip with 0.5 mg of force and moved it across the edge of the sample at 50 μm/s so that the film thickness could be deduced from the resulting height profile. Each reported value is the average of four technical replicates, one from each edge of a square sample.

### Finite element analysis

To analyze the developed strain during membrane indentation, we established a finite element model using Abaqus/Explicit. The model was axisymmetric, and cylindrical coordinates were used with radial, circumferential, and axial directions denoted by r, θ, and z, respectively. We modeled the membrane as a neo-Hookean hyperelastic material. The membrane radius, R_m_, is given as 3.175 mm based on experimental measurement. Membrane thickness was 0.056 mm for PU membranes and 0.237 mm for PDMS membranes. For more information on model geometry, refer to Supplementary Figure S1 and Supplementary Table S1 in the Supplementary Material.

We extrapolated the material properties for a PDMS elastomer of 20:1 base polymer to curing agent ratio (the ratio used by the manufacturer), using data provided by Kim and colleagues.^28^ We did not adjust the material properties for simulations of PU membranes because parametric simulations showed that the membrane strain associated with a given indentation depth did not depend on material properties (see the Supplementary Material). To simulate indentation, we translated the membrane peripheral edge (*r* = R_m_) vertically with displacement Δz = −2 mm while constraining its other degrees of freedom to be zero. We modeled the post as a fixed rigid body. General hard contact with frictions between the membrane and post were enforced in Abaqus/Explicit. We ran the simulation with explicit time integration until steady state was achieved and recorded nominal strains in radial and circumferential directions. We related the Green strain in the maximum principal direction, ɛ_G_ (the strain metric reported by the digital image correlation software, GOM Correlate), to the nominal strain in the maximum principal direction, ɛ_N_ (the value reported by Abaqus), using the following equation:

$$\varepsilon_{G}=\frac{1}{2}\left({\left(\varepsilon_{N}+1\right)}^{2}-1\right)$$

We averaged this value for the region of the top surface of the membrane within 1 mm of the center and reported it as the membrane strain.

### Stem cell culture and neuron differentiation

The iPSC line was the CS29 isogenic control cell line from Cedar Sinai. We maintained iPSCs on Matrigel (BD Biosciences, San Jose, CA) with daily feedings of mTeSR1 media (STEMCELL Technologies Inc., Vancouver, BC, Canada) and regularly passaged with Accutase (Sigma-Aldrich). Before differentiation, we passaged stem cells and plated them into mTeSR1 with 10 μM of Rho-associated, coiled-coil containing protein kinase (ROCK) inhibitor (Y-27632; DNSK International, LLC, Hamden, CT). Upon reaching ∼80% confluence, we replaced media with N2B27 medium (50% Dulbecco's modified Eagle's medium/F12, 50% Neurobasal™, supplemented with non-essential amino acid [NEAA], GlutaMAX™, N2, and B27; GIBCO, Billings, MT), containing 10 μM of SB431542 (Tocris Biosciences, Bristol, UK), 100 nM of LDN-193189 (Tocris Biosciences), 1 μM of retinoic acid (RA; Sigma-Aldrich), and 1 μM of smoothened-agonist (SAG; DNSK International). We refreshed media daily for the first 6 days. On day 6, we instead supplemented N2B27 media with 1 μM of RA, 1 μM of SAG, 5 μM of DAPT (Tocris Biosciences), and 4 μM of SU5402 (Tocris Biosciences). We fed daily until day 14. At day 14, we dissociated cultures using TrypLE Express (GIBCO), supplemented with DNase I (Worthington Biochemical Corporation, Lakewood, NJ).

We plated spinal motor neurons onto either PDMS or PU plates coated with poly-D-lysine/laminin (Life Technologies, Carlsbad, CA) and fed with neurobasal medium supplemented with NEAA, Glutamax, N2, B27, ascorbic acid (0.2 μg/mL; Sigma-Aldrich), 2% fetal bovine serum (Hyclone Laboratories, Inc., Logan, UT), and penicillin/streptomycin (Millipore, Burlington, MA). Neurobasal media additionally contained brain-derived neurotrophic factor, ciliary neurotrophic factor, and glial cell line-derived neurotrophic factor (10 ng/mL; R&D Systems, Minneapolis, MN). We transduced neurons with a lentiviral construct containing synapsin-GFP (green fluorescent protein) in suspension before plating and left in Neurobasal with 10 μM of ROCK inhibitor for the first 24 h. After the first day, we changed media to complete Neurobasal without ROCK inhibitor and refreshed half media every 3 days.

## Statistics

Statistical analysis was quantified using a two-tail ANOVA calculated in GraphPad Prism 10.

## Results

**FIG. 2.**
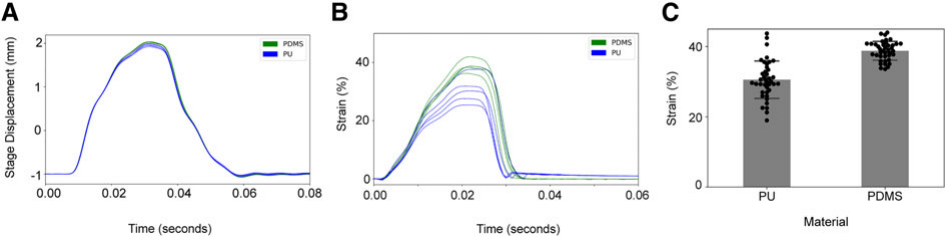
Kinematics of a 2-mm-deep indentation. (**A**) Vertical displacement of stage during indentations (*n* = 5). Note that the stage began and finished its motion 1 mm below the plate bottom. (**B**) Average membrane strain across each plate during indentation. (**C**) Peak membrane strain in each well during indentation (*n* = 40 wells, *N* = 5 plates). PDMS, polydimethylsiloxane; PU, polyurethane.

**FIG. 3.**
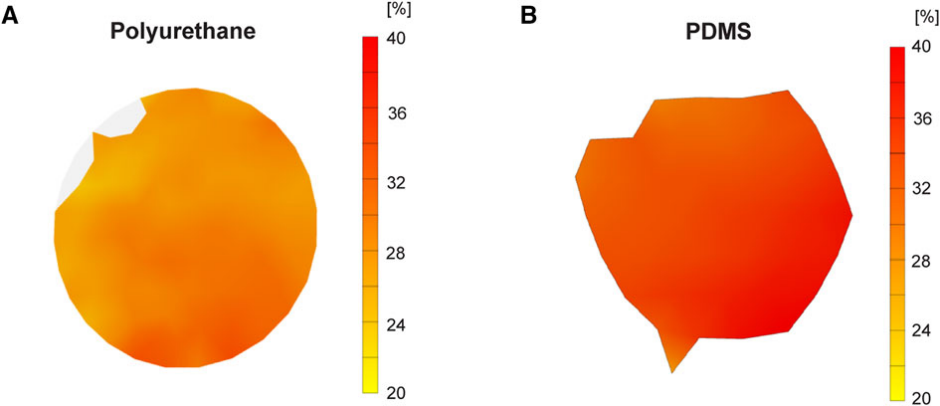
Strain distribution across the well bottom at the peak of 2-mm indentation in (**A**) a PU membrane and (**B**) a PDMS membrane. PDMS, polydimethylsiloxane; PU, polyurethane.

The stage displacement histories recorded from 2-mm-deep indentations of PDMS plates had similar shapes and peak values to those recorded from identical indentations of PU plates, although the peak values were slightly lowered in the PU case (Fig. 2A). The membrane strain histories had similar, but not identical, shapes for both materials (Fig. 2B). PDMS was completely elastic, returning to zero strain as soon as the indentation ended. By contrast, a small amount of strain persisted in the PU case after the indentation for at least a few tens of milliseconds. The peak strain was lower in the PU case than in the PDMS case (Fig. 2C). We generated heat maps of strain distribution in the center of the well at the peak of 2-mm indentation. In both membranes, strain appears to be uniformly distributed across the surface (Fig. 3A,B).

Contact profilometry showed that the average thickness of the PU membrane was 55.6 μm (standard deviation [SD] = 4.85; *n* = 8) and that of the PDMS membrane was 236.6 μm (SD = 1.42; *n* = 8). Finite element models revealed the influence of membrane thickness, membrane stiffness, and the friction at the membrane/indenter contact on membrane stretch. We confirmed that material stiffness had no effect on the peak strain value by comparing peak strain predictions from simulations run with either half or twice the actual stiffness coefficients for PDMS. Peak strains were 0.385771 and 0.385797, respectively (i.e., they were identical to the fourth significant figure), as expected based on the fact that the input (indentation depth) and output (peak strain) are both kinematic parameters. The slight difference observed beyond the fourth place of the decimal reflected the fact that the stiffer version of the model converged more quickly.

**FIG. 4.**
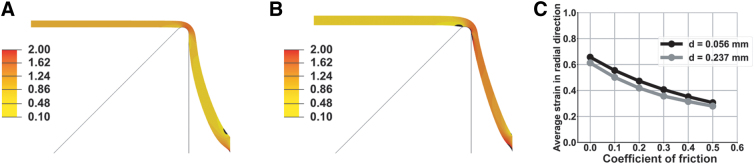
Finite element analysis of 2-mm-deep indentations in a membrane 237 μm thick. (**A**) Cross-section view of the model. The left edge is the axis of symmetry. The black outline is the profile of the indenter. The color represents the maximum principal Green strain when the coefficient of friction, μ = 0. (**B**) The same simulation as that shown in (A), except that μ = 0.5. (**C**) Variation of peak strain with membrane thickness and coefficient of friction.

Next, we ran a series of simulations to quantify influence of material thickness and the coefficient of friction, μ, on peak strain. We ran simulations with μ values ranging from 0.0 to 0.5 for both the PU membrane thickness and PDMS membrane thickness. When μ was zero, the strain in the horizontal region of the membrane inside the contact with the indenter was similar to that in the sloped region of the membrane outside the indenter (Fig. 4A). When μ was 0.5, the friction resisted the sliding of the membrane over the edge of the indenter (Fig. 4B). Therefore, the strain in the region inside the indenter contact was lower than in the zero-friction case whereas the strain outside this region was higher than in the zero-friction case. In both cases, close to the indenter contact, the strain on the top side of the membrane exceeded that on the bottom side of the membrane. However, this effect dissipated quickly as one moved away from the point of contact and the strain was constant across the thickness of the membrane throughout most of the region inside the indenter. The coefficient of friction influenced the peak strain more than the thickness across the range of values considered (Fig. 4C). We fit second-order polynomial curves to the simulation results to identify the values of μ that corresponded to the experimentally measured peak strain values (Fig. 2C) as μ = 0.253 for the PDMS membrane and μ = 0.497 for the PU membrane.

**FIG. 5.**
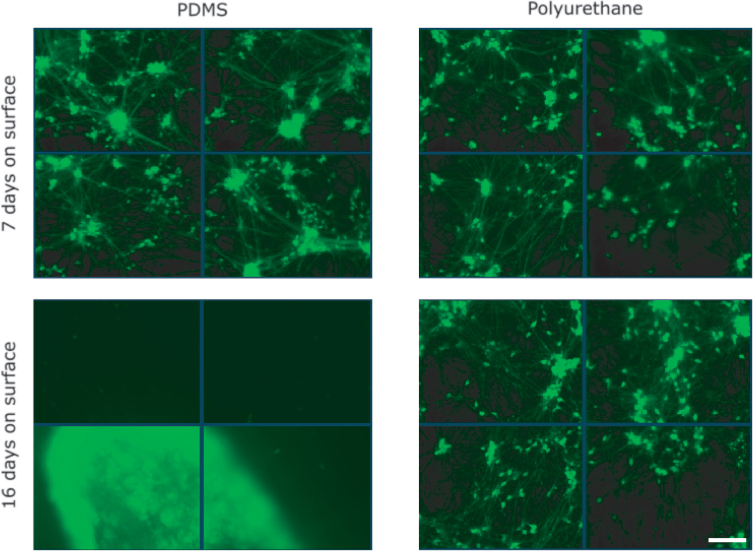
PU shows enhanced biocompatibility with reduced neuron aggregation in extended/long-term cultures. Green fluorescence = hiPSC-derived neurons plated at 75,000 cells per well and transduced with synapsin-GFP. Scale bar = 200 μm. Representative images from three repeated experiments. Nine of 10 quantifiable fields had significant aggregation in the PDMS case. Three of 12 quantifiable fields had significant aggregation in the PU case. GFP, green fluorescent protein; hiPSC, human induced pluripotent stem cell; PDMS, polydimethylsiloxane; PU, polyurethane.

Having established that PU could stretch far enough to induce a trauma phenotype in iPSC-derived neurons in an almost perfectly elastic fashion, we proceeded to test the hypothesis that cell survival on PU would exceed that on PDMS. After 16 days in culture, hiPSC-derived neurons growing on PU were healthier than identical cultures growing on PDMS. There was more aggregation and detachment in the PDMS cultures (Fig. 5). Although we still observed minor aggregation in the neuron population on PU, cells were more uniformly distributed across the surface and remained adherent. Neurons could be cultured for >22 days on PU without significant detachment or aggregation.

**FIG. 6.**
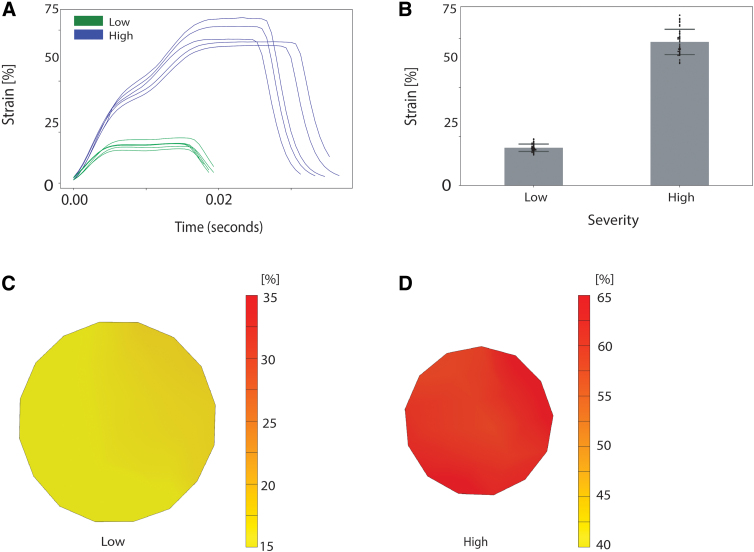
Indentation of PU membranes to depths of 1.5 mm (low) and 3.0 mm (high). (**A**) Strain histories. (**B**) Strain peaks (*n* ≥ 39 well, *N* ≥ 5 plates; error bars = standard deviations). (**C**) Representative strain field for low condition. (**D**) Representative strain field for the high condition. PU, polyurethane.

When we quantified stretch mechanics in PU membranes indented to depths of either 1.5 or 3.0 mm, we found that the peak strain for a 1.5-mm indentation was lower than that for a 2.0-mm indentation whereas the peak strain for a 3.0-mm indentation was higher (Fig. 6A,B). At the 1.5-mm depth of indentation, the strain distribution around the center of the well was uniform, whereas at the 3.0-mm depth of indentation, the strain distribution across the well bottom fluctuated across a range of 5–8% (Fig. 6C,D).

**FIG. 7.**
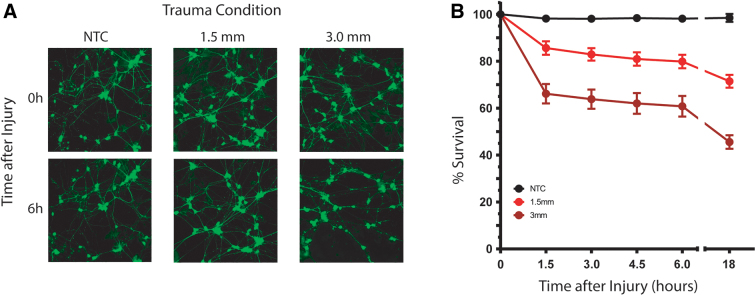
In cultures seeded on PU membranes, survival declined with increasing indentation depth. (**A**) Images of synapsin-GFP fluorescence in neurons on PU after trauma of varying levels of severity (NTC = No Trauma Condition, 1.5-mm indentation depth = mild trauma, and 3.0-mm indentation depth = severe trauma). Cells transduced with synapsin-GFP. Scale bar = 100 μm). (**B**) Cell survival over an 18-h period post-trauma. GFP, green fluorescent protein; PU, polyurethane (*p* < 0.0001 by 2-way ANOVA).

The dependence of peak strain on indentation depth in PU membranes implies that the trauma phenotype in cells cultured on these membranes will also depend on indentation depth. To test this hypothesis, we quantified survival of hiPSC-derived neurons expressing synapsin-GFP cultured on PU membranes after *in vitro* trauma. Stretching the membrane by indentation lowered survival in a dose-dependent fashion (Fig. 7A). Survival had already declined by 1.5 h after trauma, indicating that neurons degenerated quickly. Survival continued to decline up to 18 h post-trauma, the furthest time point analyzed (Fig. 7B).

## Discussion

Neurons derived from hiPSCs have been used widely with great success in the study of many important neurological conditions in recent years,^29–32^ but they have barely been used at all in the study of neurotrauma. A recent comprehensive review identified 91 *in vitro* studies of neurotrauma published between 2008 and 2018 and found that only two of these used neurons derived from hiPSCs.^17^ There are several means of traumatizing neurons in culture, but the stretch of silicone rubber cell-culture substrates is by far the most popular.^17^ Keeping hiPSC-derived neurons alive and healthy on such substrates is difficult and this challenge may explain why this cell type is not widely used in neurotrauma studies. Further progress with *in vitro* modeling of neurotrauma using hiPSC-derived neurons requires a culture substrate that is compatible with both the long-term monoculture of these cells and large elastic deformations. Our results demonstrate that PU meets these requirements.

The most important advantage of the switch to PU was the improvement in cell survival. Sustaining two-dimensional (2D) monocultures of neurons for long periods is challenging even on conventional substrates like glass and plastic. Transferring culture protocols that succeed on these substrates to stretchable substrates is non-trivial. When monolayer cultures decline, cells start to aggregate. This clumping phenomenon makes cell counting difficult or impossible, preventing analysis such as immunocytochemistry. Further, significant aggregation can be detrimental to culture health because degenerating cells are also aggregated and unable to be removed/washed away. However, the qualitative difference between cultures grown on PU and PDMS substrates was very apparent. PDMS cultures were already more clumped at day 7, and by day 16, the PDMS cultures had completely failed whereas the PU cultures remained viable (Fig. 5).

2D monocultures are sensitive to how the cell-culture substrate is coated with extracellular matrix proteins to promote adhesion. Laminin, poly-L-lysine, and polyornithine have been used for this purpose for *in vitro* models of neurotrauma.^17^ We used a single, consistent coating protocol for both PU and PDMS in this study. We did not exhaustively optimize the coating for each substrate. However, it is worth noting that the coating protocol had been optimized for PDMS^23^ and was applied to PU without modification. Therefore, it seems probable that further optimization would enhance the advantages of PU relative to PDMS.

The strain amplitude in PU membranes was lower than that in PDMS membranes when both were indented to a depth of 2 mm (Fig. 2). Strain amplitude is a key parameter because it correlates with the severity of the trauma phenotype in this model and other similar models.^16,33^ The displacement histories of the indenter apparatus were almost identical for the PU and PDMS membranes, eliminating the possibility that PU membranes deformed less because they were stiff enough to prevent the indenters from reaching the specified depth (Fig. 2A). Finite element simulations showed that the influence of thickness on membrane indentation mechanics was modest (Fig. 4C). The mechanics of bending depend sensitively on thickness, but bending effects were confined to a small domain close to the point where the indenter contacted the membrane (Fig. 4A,B). On the other hand, the coefficient of friction, μ, strongly influenced membrane strain because friction resists the motion of the membrane across the edge of the indenter. We found that μ was higher for PU than for PDMS.

However, it is worth noting that μ values are not intrinsic to the materials, but instead depend on both the material properties, the Teflon coating on the indenter, and the lubricant (corn oil). We were reluctant to use potentially toxic lubricants with PDMS culture substrates because PDMS is absorbent, so cells growing on the top of the membrane could be exposed to substances applied to the bottom of the membrane. PU might permit a wider range of options with respect to lubrication.

Another difference between the stretch mechanics of the PU and PDMS membranes was the way they recovered from indentation. The strain in the PDMS membrane returned to zero at the end of the indentation event. In the PU membrane, the strain returned to zero and then rebounded slightly to a residual strain of 1–2%. This residual strain appears to dissipate quickly. Almost half of it resolved in the 30-ms time window subsequent to the indentation events in Figure 2B. Strains of >10% are required to induce injury *in vitro*,^33^ so these residual strains are not particularly noteworthy in the context of a single injury model. If PU membranes are to be used in a repeated injury model, it would be worthwhile to confirm that these residual strains resolve completely between repeated stretch events so that initial conditions remain consistent.

The injury phenotype observed in hiPSC-derived neurons grown on PU substrates was dose dependent and progressed over time (Fig. 7). The capacity to observe progressive post-traumatic pathology in these cultures bodes well for their application to questions about why persons with certain genotypes have worse outcomes than others after apparently similar neurotrauma events. This question is one of the most important in the field. Clinical trials in TBI and SCI remain extremely difficult, in part, because of the wide variation among patients within the same treatment group.^34,35^ Understanding genetic factors influencing the response to neurotrauma will facilitate stratifying these trials into groups with similar probable outcomes and make it easier to detect therapeutic effect. Genotype-trauma interactions will also provide clues about the molecular mechanisms of pathology.

This study is subject to several limitations. We used a single phenotype—cell survival—to demonstrate a dose-dependent response to trauma, but there are many other important phenotypes that remain to be explored. Only neurons were considered in this study. Astrocytes, microglia, and epithelial cells can also be generated from hiPSCs and also play important roles in neurotrauma pathology. However, these cell types are generally easier to culture than neurons, so that conditions that succeed for neurons are likely to succeed for them, too. On a related note, survival of neuronal monolayer cultures can be improved by seeding them onto a layer of supportive cells, such as rodent glia. This strategy may succeed on stretchable substrates. However, it complicates some outcomes, such as RNA sequencing, that are more difficult to interpret when multiple cell types are present, so a practical strategy for neuron monoculture remains valuable. In summary, we found that PU is a viable alternative to PDMS for *in vitro* models of neurotrauma that dramatically enhances the survival of hiPSC-derived neurons, making it easier to apply the unique and powerful experimental approaches enabled by this cell source to neurotrauma pathology.

## Supplementary Material

Supplemental data

## Abbreviations Used

2D: two-dimensional
ALS: amyotrophic lateral sclerosis
GFP: green fluorescent protein
hiPSC: human induced pluripotent stem cell
iPSC: induced pluripotent stem cell
NEAA: non-essential amino acid
PDMS: polydimethylsiloxane
PU: polyurethane
RA: retinoic acid
ROCK: Rho-associated, coiled-coil containing protein kinase
SAG: smoothened-agonist
SCI: spinal cord injury
SD: standard deviation
TBI: traumatic brain injury
