# Supplementary material for: Polyurethane Culture Substrates Enable Long-Term Neuron Monoculture in a Human in vitro Model of Neurotrauma
Source: Neurotrauma Rep. 2023 Oct 16;4(1):682–92. doi: 10.1089/neur.2023.0060 (PMC10615064; doi:10.1089/neur.2023.0060)
Supplement: Supplemental data [file Suppl_Data.docx]

**Supplementary Material: Polyurethane culture substrates enable long term neuron monoculture in a human in vitro model of neurotrauma**

Angela Mitevska^1†^, Citlally Santacruz^1†^, Eric J. Martin^2^, Ian E. Jones^3^, Arian Ghiacy^3^, Simon Dixon^4^, Nima Mostafazadeh^1^, Zhangli Peng^1^, Evangelos Kiskinis^2,5^, John D. Finan*^3^

^1^Department of Biomedical Engineering, University of Illinois at Chicago, Chicago, IL, USA

^2^The Ken & Ruth Davee Department of Neurology, Northwestern School of Medicine, Feinberg School of Medicine, Chicago, IL

^3^Department of Mechanical and Industrial Engineering, University of Illinois at Chicago, Chicago, IL, USA

^4^Biomer Technology Ltd., Warrington, United Kingdom.

^5^Department of Neuroscience, Northwestern University Feinberg School of Medicine, Chicago, IL, 60611, USA

†These authors contributed equally to this work.

*Corresponding author

Contact information for corresponding author:

Email: [jdfinan@uic.edu](mailto:jdfinan@uic.edu)

Phone: +1 312 413 1031

Address: 842 W. Taylor

ERF – Rm 2039

Chicago, IL 60607

Fax: (312) 413-0447

**Supplementary Methods for Finite Element Modeling Study**:

The coefficients of the neo-Hookean constitutive model for the membrane were C_0_ = 0.2684 MPa and bulk modulus = 496.12 MPa, where C_0_=0.5 μ and μ is the shear modulus. To confirm that these coefficients had no influence on the strain near the center of the membrane in a simulation of a 2 mm deep indentation, we ran the simulation with these coefficients doubled (C_0_ = 0.5368 MPa, Bulk modulus = 992.24 MPa) and then ran it again with these coefficients halved. (C_0_ = 0.1342 MPa, Bulk modulus = 248.06 MPa).


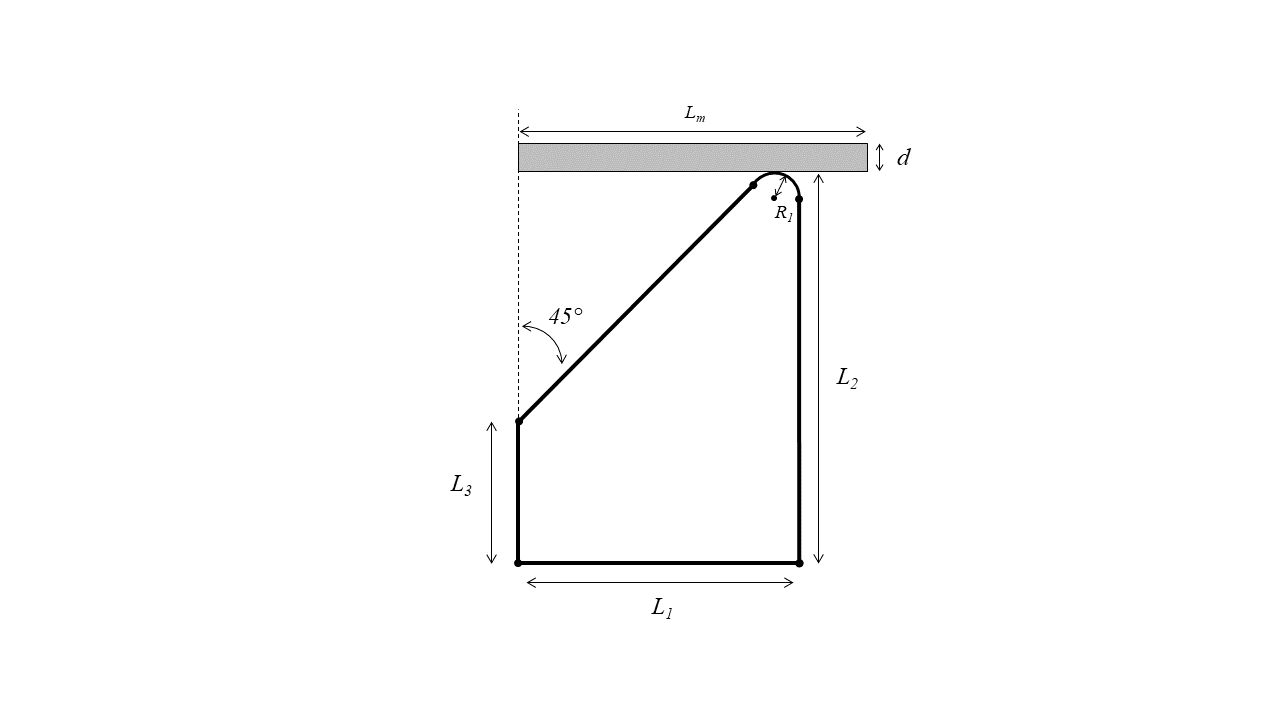


**Figure S1:** Geometry of finite element model

**Table S1:** Properties of finite element model

| Post geometry | L_1_ | 2.52 mm |
| --- | --- | --- |
|  | L_2_ | 5.04 mm |
|  | L_3_ | 0.15 mm |
|  | R_1_ | 0.1 mm |
| Membrane geometry | L_m_ | 3.175 mm |
|  | D | 0.056 and 0.237 mm |
| Membrane properties | Μ | 536.8kPa |
|  | K | 496.1 MPa |
